# Host-parasite Responses Outcome Regulate the Expression of Antimicrobial Peptide Genes in the Skin of BALB/c and C57BL/6 Murine Strains Following *Leishmania major* MRHO/IR/75/ER Infection

**Published:** 2018

**Authors:** Hamid DANESHVAR, Amir TAVAKOLI KARESHK, Iraj SHARIFI, Alireza KEYHANI, Razieh TAVAKOLI OLIAEE, Arash ASADI

**Affiliations:** 1. Dept. of Immunology, School of Medicine, Kerman University of Medical Sciences, Kerman, Iran; 2. Infectious Disease Research Center, Birjand University of Medical Sciences, Birjand, Iran; 3. Dept. of Parasitology and Mycology, School of Medicine, Kerman University of Medical Sciences, Kerman, Iran; 4. Leishmaniasis Research Center, Kerman University of Medical Sciences, Kerman, Iran

**Keywords:** Antimicrobial peptides, Cathelicidin, Cathelin-related antimicrobial peptide, Cytokine, Defensin, *Leishmania major*, Mouse β-defensin

## Abstract

**Background:**

Different background of immunity responses determine resistance or susceptibility of certain mouse strains to *Leishmania major* infection. Some have been well known previously. Antimicrobial peptides (AMPs) such as cathelicidins and defensins are unique fragments of innate immunity system with well-known effects against the invasion pathogens. Despite their outstanding roles and being of extensive cases of cutaneous leishmaniasis (CL) caused by *L. major*, they have been less studied in *Leishmania* fields. The aim of present study was to determine whether these components play a role in the protection of skin against *Leishmania* infections.

**Methods:**

The animal model of *Leishmania* infection was established by the subcutaneous inoculation of 5×10^6^(parasites/ml) from the stationary phase of *L. major* promastigotes to BALB/c and C57BL/6 mice from January 2016 to August 2016 in Kerman Province, southeast of Iran. After 1, 3 and 7 d of post-infection (PI), the samples needed for detecting of the mRNA levels of mouse beta-defensin (mBD)-1, mBD2, mBD3, mBD4, mBD6, cathlin-related antimicrobial peptide (CRAMP), interleukin (IL)-10, IL-12 and parasite load were taken under standard methods.

**Results:**

The findings related to cytokines profiles in BALB/c (↑IL-10, ↓IL-12) and C57BL/6 mouse strains (↓IL-10, ↑IL-12) demonstrated that immunity system has been accurately activated during CL caused by *L. Major* parasites. We also observed a significant up-regulation of all aforementioned AMPs genes in BALB/c mice at selected times compared to another strain.

**Conclusion:**

CL occurred in BALB/c mice in spite of the fact that the expression of AMPs was higher than the other strain. AMPs genes are well expressed to provide defense against the parasites that have increased and escaped from immunity system but cannot create an absolute protection.

## Introduction

Leishmaniasis is a vector-borne disease created by intracellular protozoan parasites of the *Leishmania* genus (1). Three clinical complications such as cutaneous (CL), mucocutaneous (MCL) and visceral (VL) have been recognized (2). The suitable mammals including humans are commonly infected by this intracellular parasite (3).

Infection occurs when an infected female sand-fly inoculates the stationary phase of promastigotes into the dermis of a suitable vertebrate host(4). It is recognized as a serious health problem in many developed and developing countries (5). Approximately 0.2–0.4 million VL and 0.7–1.2 cases of CL occur each year (1). *L. major* discussing in the present study is endemic in many countries such as Iran (6).

Some fragments of immunity system such as cytokines play important roles during *Leishmania* infection. Pathways leading to IL-12 and interferon gamma (IFN-γ) cause the protection against *Leishmania* infection in C57BL/6 mice (7, 8). Instead, the increase of IL-10 and IL-4 predispose BALB/c mice to infection (9, 10). However, having no effective vaccine, safe drugs, emergence of resistant strains and immunodeficiency syndromes impel us to search for new knowledge such as antimicrobial peptides (AMPs).

Historically, gramicidin was the first AMPs separated from a soil *Bacillus* strain (11). More than 5000 AMPs have been illustrated so far (12). They are unique compounds of innate immunity and resistance to them is rare (13). They classified into subgroups on the basis of their amino acid composition and structure (14). Cathelicidins and defensins consist the main groups of them (15, 16). Cathelin-related antimicrobial peptide (CRAMP) is the only cathelicidin found in mouse strains, while several genes for defensins including mouse beta-defensin (mBD) have been known (17, 18). They directly participate for restriction of infections or indirectly with modulation of immunity responses (19, 20).

However, despite being remarkable cases of CL, little studied is found about AMPs role in *Leishmania* infections. Therefore, the major aim focused to show whether these compounds influence susceptibility or resistance to *L. major* infection.

## Materials and Methods

**Animals**: Forty-eight BALB/c and C57BL/6 mice (inbred sex-age matched, 6 to 8 wk old, weight from 20 to 24 gr) were purchased from Animal Facility Center of Razi Institute (Karaj, Iran) in 2016. Animals were kept under suitable conditions and handled based on standard protocols in line with the guideline of Kerman University of Medical Science.

**Parasite**: *L. major* promastigotes strain MRHO/IR/75/ER (Iranian type collection) was cultured in 50 ml flask containing RPMI 1640 (Biosera, UK) enriched with 10% heated inactivated fetal bovine serum (HIFBS) and 1% pen/strep antibiotics (Biosera, UK).

**Animal challenge:** For the experiments, each mouse strain was randomly divided into two groups: uninfected (control), challenged by *L. major* (infected). The challenge groups of both strains (n=8 mice/group/time point) were inoculated with the stationary phase of *L. major* promastigotes (5×10^6^ parasites/ml) via the subcutaneous route in the base of their tails (as infected group) compared to control group which received no parasites and kept under specific conditions for 1, 3 and 7 days.

### Samples collection

To detect the AMPs gene expression and parasite load, skin biopsies were taken for all groups from the site of inoculation at selected times and kept under suitable conditions. To analyze the cytokine genes expression, macrophages were isolated from peritoneal cavity of all infected and control groups at selected times of PI as previously described (21). The cells were placed in Dulbecco's Modified Eagle's Medium (DMEM) enriched with 10% HIFBS and 1% pen/strep antibiotics. The non-adherent cells were removed and adherent cells (macrophage cells) were gathered for the detection of cytokine profiles.

### Ethical approval

To work on animals, we got permission number ir.kmu.rec.1394.208 from the Ethical Board of Kerman University of Medical Science (Kerman, Iran).

### AMPs and cytokine genes expression assay

For analysis of AMPs and cytokine genes expression, total RNA was extracted from the certain specimens of all infected and control groups using RNA Purification kit (Jena Bioscience, Germany) and quantified by a NanoDrop 2000 spectrophotometer (Thermo Scientific, Wilmington, DE).

Three microgram (μg) was transcribed to complementary DNA (cDNA) using Accu-Power® CycleScript RT PreMix (dN6) random primer (Bioneer, Korea). Briefly, 3 μg of RNA was adjusted in 20 μl DEPCI-DW and totally added to each lyophilized tube. Thermal profile was performed as follows: 12 cycles (20 °C for 30 sec, 42 °C for 4 min, 55 °C for 30 sec) and 95 °C for 5 min.

**Fig. 1:**
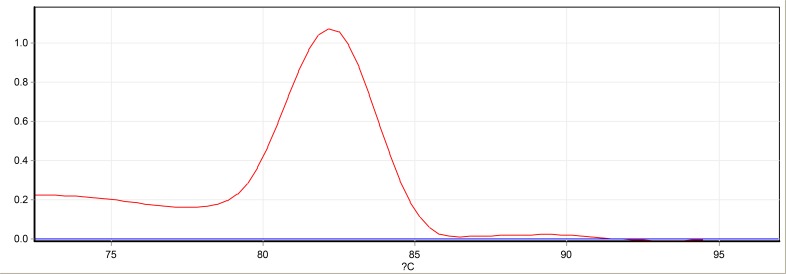
Melt curve from qPCR of mBD1gene of one reaction

Relative quantitative real-time PCR was utilized using a Rotor GENE Q (Qiagen, Germany). B2M and RPII were used to amplify house-keeping cDNA. The specific primers were applied to amplify desirable amount of genes cDNA (Table 1). Briefly, the 15μl of each reaction mixture (1μl cDNA, 7 μl SYBR Green, 5μ1 DW, 1μl primer forward 2.5 Pmol, 1μL primer reverse 2.5 Pmol) was prepared using SYBR Premix EX Taq2 Master Mix (Takara, Japan). Thermal profile was performed as follows: 95 °C for 1 min, 40 cycles (94 °C for 15 sec, 58 °C for 30 sec, 72 °C for 20 sec). For mBD1 gene, melt curves were analyzed for other genes to evaluate the quality of SYBER green and specific products. All data were analyzed by 2^−ΔΔCT^ method (22) (Fig. 1).

**Table 1:** Primer sequences

***Template***	***Forward and reverse sequences (5′-3′)***
mBD1	F-AGGTGTTGGCATTCTCACAAG
	R-GCTTATCTGGTTTACAGGTTCCC
mBD2	F-ATACGAAGCAGAACTTGACCACTG
	R-AATCATTTCATGTACTTGCAACAGG
mBD3	F-GCATTGGCAACACTCGTCAGA
	R-CGGGATCTTGGTCTTCTCTA
mBD4	F-GCAGCCTTTACCCAAATTATC
	R-ACAATTGCCAATCTGTCGAA
mBD6	F-GTTCATGCAATGGAGGTTTTCG
	R-TTTTCTGCGGCAGCATCTGAT
mBD14	F-TCCAGGGGACGCATTCCTA
	R-ACCGCTATTAGAACATCGACCTA
CRAMP	F-GGCTGTGGCGGTCACTAT
	R-GTCTAGGGACTGCTGGTTGAA
RV1	F-CTTTTCTGGTCCCGCGGGTAGG
	R-CCACCTGGCCTATTTTACACCA
IL-10	F-GCTGGACAACATACTGCTAACC
	R-ATTTCCGATAAGGCTTGGCAA
IL-12b	F-TGGTTTGCCATCGTTTTGCTG
	R-ACAGGTGAGGTTCACTGTTTCT
RPII	F-CTACACCACCTACAGCCTCCAG
	R-TTCAGATGAGGTCCATGAGGAT
B2M	F-TTCTGGTGCTTGTCTCACTGA
	R-CAGTATGTTCGGCTTCCCATTC

### Detection of parasite burden

First, we extracted DNA from 10^6^ parasites/ml of *L. major* promastigotes strain MRHO/IR/75/ER using QIAamp DNA Mini Kit (Qiagen, Germany) and serial dilutions of DNA ranging from 10^6^ to 10^1^ per reaction were utilized for preparation of standard curve.

In parallel, we extracted DNA from 20 mg of each skin biopsy by Primeprep Genomic DNA Isolation Kit from Tissue (GeNet Bio, Korea) from all infected mice. The RV_1_ and RV2 primers were applied to amplify a 145 bp sequence from kinetoplast DNA (kDNA) minicircles of *Leishmania* genus (23, 24), and are conventionally used for diagnostic purposes. In the present study, the mentioned primers were used for the detection of *L. major* kDNA copy numbers using real-time PCR method.

**Fig. 2:**
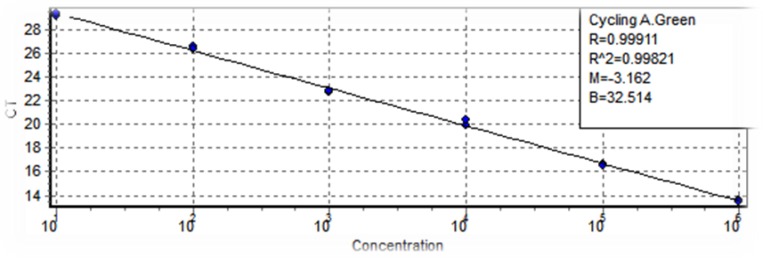
Standard curve. Amplification of kDNA from *L. major* with serial dilutions ranging from 10^6^ to 10^1^ parasites per reaction

The 15μl reaction mixture contained (6μl DW, 6μl SYBR Green, 1μl RV_1_ primer forward 2.5 Pmol, 1μl RV_1_ primer reverse 2.5 Pmol, 1μl DNA template) was used for each sample in real-time PCR. The thermal profile was as follows: 95 °C for 1 min, 40 cycles (94 °C for 15 sec, 56 °C for 30 sec). The parasite loads automatically obtained compared to standard curve (Fig. 2).

## Results

### Statistical analysis

Our findings were exhibited as mean ± SEM and their analysis was carried out using SPSS statistical software version 17.0 (SPSS Inc, Chicago, IL, USA). Student’s *t*-test was applied to obtain differences between the groups. Moreover, *P*<0.05 was considered as significant level.

### Antimicrobial peptides gene expression

Relative quantitative real-time PCR was applied to measure the mRNA levels of defined AMPs following *L. major* infection. First, the expression levels of selected genes of infected groups were separately determined against their controls in both strains.

**Fig. 3:**
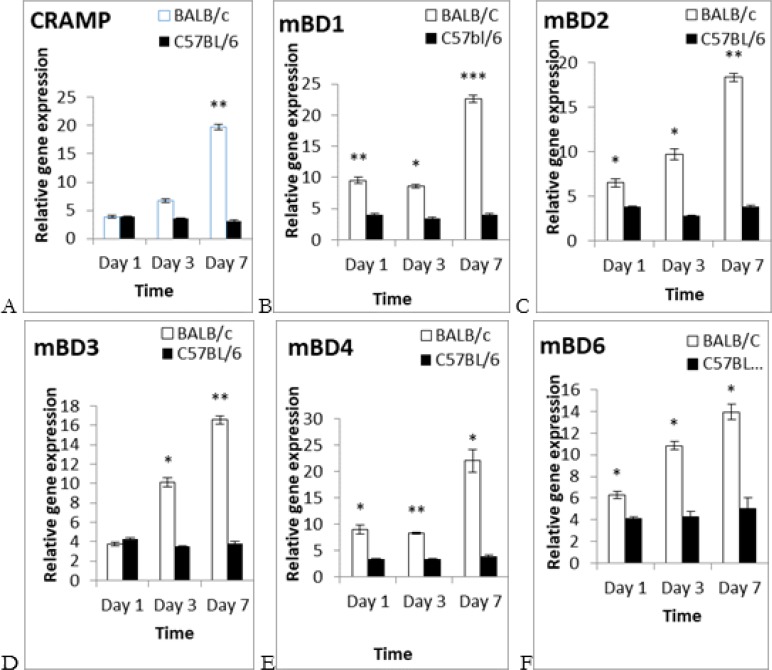
**Different AMPs gene expression in mouse strains after infection with *L. major* parasites**. BALB/c and C57BL/6 mice were infected by the stationary phase of *L. major* promastigotes at the base of their tails. Skin samples were taken from both strains. The expression levels of AMPs were separately detected against their controls for both strains. The obtained results in the BALB/c mice (white bar) compared to the C57BL/6 mice (black bar) at selected times. (A) CRAMP expression. (B) The expression levels of mBD1. (C) The mBD2 gene expression. (D) The mBD3 expression. (E) The mRNA levels of mBD4. (F) The mBD6 gene expression. Data were shown as mean ± SEM and *P*<0.05 considered as significance level

Finally, the obtained results compared between both strains. There was an up-regulation of all AMPs at first day in both mouse strains against their controls. This up-regulation continued for all AMPs in BALB/c mice for day 3 and 7 PI, while nearly remained stable in the C57BL/6 mice (data have not been shown). Comparing strains showed that the expression level of CRAMP was no significant on day 1 and 3 of PI. However, our results showed a significant up-regulation of CRAMP in the BALB/c mice (19.71±0.48) in comparing to C57BL/6 mice (3.13±0.38) on day 7 PI (Fig. 3A).

The significance expression levels of mBD_1_ in BALB/c were (9.53±0.46), (8.60±0.29), (21.86±0.53) compared to (4±0.19), (3.49±0.12), (3.96±0.26) in the C57BL/6 mice on day 1, 3 and 7 of PI respectively (Fig. 3B). We found significant up-regulation of mBD_2_ (6.53±0.47), (9.74±0.60), (17.76±0.430) for the BALB/c mice in contrast to (3.86±0.09), (2.83±0.10) and (3.91±0.10) for the C57BL/6 at selected times of PI (Fig. 3C). Moreover, the significance levels of mBD3 in BALB/c mice were (10.14±0.47), (16.57±0.40) versus to (3.48±0.09), (3.83±0.15) in C57BL/6 mice only on day 3 and 7 of PI (Fig. 3D).

In addition, there were significant differences in mBD4 gene expression in BALB/C in comparing to C57BL/6 mice at all times. The expression levels of mBD4 in the BALB/c mice were (8.96±0.87), (8.39±0.19), (22.06±2.1) in contrast to (3.36±0.08), (3.41±0.13), (3.96±0.26) in the C57BL/6 mice on day 1, 3 and 7 of PI respectively (Fig. 3E). Finally, there was significant differences of mBD6 from ranging (6.29±0.30), (10.84±0.38), (13.96±0.68) for BALB/c mice to (4.14±0.10), (4.29±0.51), (5.07±0.92) for the C57BL/6 mice on day 1, 3 and 7 of PI (Fig. 3F).

### Cytokine gene expression

Cytokine expression was assessed on macrophage cells of all infected and controls mice using relative quantitative real-time PCR method. The expression levels of the selected cytokines in the infected groups were determined against their controls for both strains. Finally, the results in BALB/c mice were compared with the other strain.

**Fig. 4:**
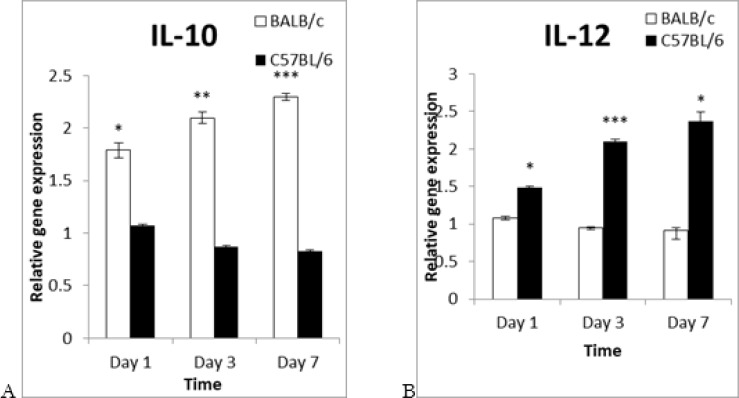
**Detection of cytokine expression.** BALB/c and C57BL/6 mice were infected by the stationary phase of *L. major* promastigotes at the base of their tails. Macrophages were isolated from both strains at selected times of PI. The expression levels of cytokines were separately detected against their controls for both strains. The expression levels of IL-10 and IL-12 in BALB/c mice (white bar) were compared to C57BL/6 mice (black bar). (A) IL-10 expression. (B) IL-12 expression. Data were expressed as mean ± SEM and *P*<0.05 considered as significant level

The significance levels of IL-10 in BALB/c mice were (1.79±0.079), (2.09±0.057), (2.29±0.030) in contrast to (1.07±0.014), (0.87±0.011) and (0.83±0.010) in C57BL/6 mice on day 1, 3 and 7 of PI respectively (Fig. 4A). Instead, C57BL/6 mice significantly showed the expression of IL-12 from ranging (1.48± 0.019), (2.14±0.082) and (2.37±0.360) versus to (1.07 ± 0.029), (0.955±0.012), (0.916±0.035) in the BALB/c mice on day 1, 3 and 7 of PI (Fig. 4B).

### Parasite burden

We detected the parasite burden in skin biopsies of both infected strains by quantitative real-time PCR using standard curve. In BALB/c mice, the parasites burden increased with an ascendant trend so that the highest level was observed on day 7 of PI, while these processes were completely reverse in C57BL/6 mice (Table 2).

**Table 2:** Parasites burden in the mice skin at selected times PI

***Sample***	***Time***	***Number (×10^3^) BALB/C***	***Percent***	***Number (×10^3^) C57BL/6***	***Percent***
Skin	24h	3.957	20.3	4.434	55.5
Skin	72h	7.466	38.3	2.203	27.7
Skin	7Day	8.057	41.4	1.343	16.8

## Discussion

Leishmaniasis is a serious problem of many countries (25), and nearly 1–1.5 million new cases are added each year (26). It takes enormous economic burden annually (27). *Leishmania* resistance genes may have role in the severity of the disease (28). Also, Prior studies have characterized that host-pathogen responses are the most important criteria for resistance or susceptibility to *L. major* infection.

Hence, we selected mice from two different strains, which genetically show various responses to infection. *L. major* produces skin lesions in BALB/c mice as susceptible strain, while inhibits in the C57BL/6 as resistant strain (29). The finding measured for cytokine profiles revealed that immunity system has accurately responded to infection in both mouse strains (Fig. 4A–B). Produced interleukin (IL)-12 by different kinds of cells such as macrophages differentiate nave lymphocytes to T helper (T h)-1cells (30). This cell can express and produce some cytokines such as interferon gamma (IFN-γ), tissue necrosis factor (TNF)-α and IL-12 which are necessary for promoting of immunity system toward cellular immunity and activating of macrophage cells (31).

Activated macrophages by mentioned cytokines especially IFN-γ express inside inducible nitric oxide and nitric oxide synthetase2 (NOS2) enzymes which produce (NO) from their substrate and kill the parasites inside themselves. In contrast, IL-10 tends nave lymphocytes differentiate to Th_2_ cell which produces plenty of cytokines including IL-4, IL-5, IL-7and IL-10, which activate humoral system and cause susceptibility to *L. major* infection in BALB/c mice (32).

Next step, we investigated AMPs gene expression to show whether they alter in both mouse strains after challenging with *L. major* parasites. We measured high levels of AMP genes expression in BALB/c mice compared to the other strain (Fig. 3). It was contrary to our hypothesis. The cells have ability to respond to stimuli from external or internal of their environments (33).

Most often, pathogens act as positive inducer and enhance the expression of genes. Consequently, we measured the parasite burden in both mouse strains. We observed that the parasite burden was significantly higher in BALB/c skin compared to C57BL/6 mice. Actually, the immune responses in BALB/c mice are inadequate following *Leishmania* infection and cause the parasites escape from immune system and proliferate at the site of inoculation, which ultimately induces gene expression more in contrast to the other strain. Instead, effective immune responses destroy or inhibit from the reproduction of parasites, which finally lead to reduced gene expression of AMPs in the resistant mice. Our results completely synchronized with other studies (34).

## Conclusion

The host-parasite response outcome regulate the skin AMPs gene expression following *L. major* infection.
